# Reply: *In vitro* and *in vivo* anticancer efficacy of unconjugated humanised anti-CEA monoclonal antibodies

**DOI:** 10.1038/sj.bjc.6604549

**Published:** 2008-08-12

**Authors:** S Q Ashraf, P J Conaghan, J L Wilding, W F Bodmer

**Affiliations:** 1 Cancer and immunogenetics laboratory, Weatherall Institute of Molecular Medicine, John Radcliffe Hospital, University of Oxford, Headley Way, Oxford OX3 9DS, UK

**Sir,**

In response to Blumenthal *et al*, we would like to further clarify a few points that have been raised. The main one relates to the statement made in our paper that ‘there are so far no unconjugated or ‘naked’ antibodies to carcinoembryonic antigen (CEA) being used for the treatment of colorectal cancer’ (Conaghan *et al*, 2008). Blumenthal *et al* suggest that this is not correct; however, they fail to provide evidence to the contrary in their letter. They quote three published articles in support of their argument. However, the first one relates to a targeting study using MN-14 (Sharkey *et al*, 1995). In the 1980s, radiolabelled murine PR1A3 was also demonstrated to be highly specific for human colorectal lesions (Granowska *et al*, 1989). The other two papers present data relating to the use of a radioconjugate form of MN-14 (Hajjar *et al*, 2002; Liersch *et al*, 2005). In the letter from Blumenthal *et al*, there is reference to unpublished results with unconjugated MN-14 being used in patients. It is however difficult to make an informed response regarding unpublished data. Thus, to our knowledge, the point made in our paper still holds true: no unconjugated antibody that targets CEA has been licensed in the treatment of colorectal cancer in humans by the clinical licensing authorities in the United Kingdom or United States. This, of course, includes MN-14.

We acknowledge the *in vitro* and preclinical work that has been published using MN-14 (labetuzumab), which has been followed with interest over the years. A reference is actually made to this antibody in the introduction of our paper (Liersch *et al*, 2007; Conaghan *et al*, 2008). On a broader note, there are in fact over 200 antibodies that are under clinical testing in oncology (Reichert and Valge-Archer, 2007). Eight of these use CEA as a target, including T84.66, which, like MN14, have been used in clinical trials as radioconjugates (Wong *et al*, 2004; Reichert and Valge-Archer, 2007). Our paper certainly did not try and create an impression that PR1A3 was the only antibody to target CEA.

It is interesting that 8 out of the 12 antibodies that are currently licensed for therapy in oncology are unconjugated, and that there are no conjugated antibodies licensed for therapy in solid tumours (Carter, 2006; Reichert and Valge-Archer, 2007). This may be a result of the poor outcomes in clinical trials of radioconjugates of murine antibodies in the 1980s. Conjugating antibodies to radioisotopes introduces the problem of bystander damage, complex technology involved in conjugation and the issue of adequate radiation delivery into solid tumours (Goldenberg, 2002; Sharkey and Goldenberg, 2005; Reichert and Valge-Archer, 2007).

MN-14 has been used in preclinical studies. However, xenografts have the inherent problem of being a poor comparative model for antibody efficacy in humans (Wilkinson *et al*, 2001). This is based on two factors, abnormal vascularity of xenografts as well as the immunodeficient nature of the animals. Furthermore, there are differences in the murine and human immune system, which further complicates the matter, thereby raising major concerns about drawing parallels with what happens in humans. It is interesting that in the xenograft model, MN-14 was only effective in the GM-CSF treated group (Blumenthal *et al*, 2005). This cytokine is known to stimulate monocytes and promote their differentiation into macrophages. In mice, this cell type expresses Fc*γ*IV, which is homologous to Fc*γ*III in humans (Nimmerjahn *et al*, 2005). A better *in vivo* model, which may better reflect antibody targeting, is a spontaneous tumour model in which immunocompetent MIN mice develop CEA-positive tumours (Wilkinson *et al*, 2001). We are currently in an advanced stage of testing unconjugated murine PR1A3 in this model.

We feel that Blumenthal *et al* have failed to understand the main message of our paper, which relates to the importance of immune-mediated antibody responses. The emergence of immune-based mechanisms has become increasingly appreciated (Carter, 2006; Clynes, 2006). The results in our paper show that humanised IgG1 PR1A3 is able to elicit antibody-dependent cellular cytotoxicity (ADCC) against a range of human colorectal cancer cell lines using human effector cells (Conaghan *et al*, 2008). This is in agreement with the previous findings that MN-14 is able to trigger ADCC of CEA-positive colorectal cell lines, LoVo and LS174T (Blumenthal *et al*, 2005). Our study further defines NK cells as an important effector cell type in eliciting this response in humans. Significantly, PR1A3-induced NK-cell-mediated killing of colorectal cancer cells is not inhibited by free CEA, which is an important characteristic for any anti-CEA antibody to be successful *in vivo*. This can be explained by the specific binding of PR1A3 to membrane-bound CEA. Previous work has identified the B3-GPI anchor of CEA as being the epitope of PR1A3 (Durbin *et al*, 1994; Stewart *et al*, 1999). The authors feel that this information can be used to further engineer PR1A3 for maximal clinical effectiveness in humans. Like Blumenthal *et al*, we would envisage this happening in partnership with current chemotherapeutic regimens.
